# Illegitimate recombination: An efficient method for random mutagenesis in *Mycobacterium avium* subsp. *hominissuis*

**DOI:** 10.1186/1471-2180-12-204

**Published:** 2012-09-11

**Authors:** Faisal Asghar Khattak, Ashutosh Kumar, Elisabeth Kamal, Ralph Kunisch, Astrid Lewin

**Affiliations:** 1 Robert Koch-Institute, Division 16 Mycology/Parasitology/Intracellular Pathogens, Nordufer 20, Berlin 13353, Germany; 2 Faculty of Biological Sciences, Islamia College Peshawar (a public sector University), KhyberPakhtunkhwa, Pakistan; 3 Pathogen Biology Laboratory, Department of Biotechnology, School of Life Sciences, Unversity of Hyderabad, Hyderabad, India

**Keywords:** Mycobacterium, *Mycobacterium avium* subsp. *hominissuis*, Non-tuberculous mycobacteria, Virulence, Mutagenesis, Illegitimate recombination

## Abstract

**Background:**

The genus *Mycobacterium* (*M*.) comprises highly pathogenic bacteria such as *M. tuberculosis* as well as environmental opportunistic bacteria called non-tuberculous mycobacteria (NTM). While the incidence of tuberculosis is declining in the developed world, infection rates by NTM are increasing. NTM are ubiquitous and have been isolated from soil, natural water sources, tap water, biofilms, aerosols, dust and sawdust. Lung infections as well as lymphadenitis are most often caused by *M. avium* subsp. *hominissuis* (MAH), which is considered to be among the clinically most important NTM. Only few virulence genes from *M. avium* have been defined among other things due to difficulties in generating *M. avium* mutants. More efforts in developing new methods for mutagenesis of *M. avium* and identification of virulence-associated genes are therefore needed.

**Results:**

We developed a random mutagenesis method based on illegitimate recombination and integration of a Hygromycin-resistance marker. Screening for mutations possibly affecting virulence was performed by monitoring of pH resistance, colony morphology, cytokine induction in infected macrophages and intracellular persistence. Out of 50 randomly chosen Hygromycin-resistant colonies, four revealed to be affected in virulence-related traits. The mutated genes were *MAV_4334* (nitroreductase family protein), *MAV_5106* (phosphoenolpyruvate carboxykinase), *MAV_1778* (GTP-binding protein LepA) and *MAV_3128* (lysyl-tRNA synthetase LysS).

**Conclusions:**

We established a random mutagenesis method for MAH that can be easily carried out and combined it with a set of phenotypic screening methods for the identification of virulence-associated mutants. By this method, four new MAH genes were identified that may be involved in virulence.

## Background

The genus *Mycobacterium* (*M*.) comprises highly pathogenic bacteria such as *M. tuberculosis* as well as environmental opportunistic bacteria called NTM. They are ubiquitous and have been isolated from soil, natural water sources, tap water, biofilms, aerosols, dust and sawdust [1-3]. Remarkably, NTM are resistant to amoeba and protected against adverse conditions inside amoebal cysts [4]. While the incidence of tuberculosis is declining in the developed world, infection rates by NTM are increasing [5]. NTM cause skin infections, lung diseases, lymphadenitis and disseminated disease mostly in immuno-compromised persons [5]. Lung infections as well as lymphadenitis are most often caused by *M. avium*[5,6], and *M. avium* is considered to be among the clinically most important NTM [7].

*M. avium* can be divided into four subspecies. *M. avium* subsp. *paratuberculosis* (MAP) causes the Johne’s disease in ruminants; *M. avium* subsp. *avium* (MAA) and *M. avium* subsp. *silvaticum* infect birds; and finally *M. avium* subsp. *hominissuis* (MAH) which causes disease in humans [8].

The main route of infection in AIDS patients is the invasion of mucosal epithelial cells of the gastrointestinal tract, while in non-AIDS patients infections mainly occur through the respiratory route [9]. Recognition of *M. avium* by mouse macrophages involves binding of a 20 – 25 kDa lipoprotein from the cell envelope of *M. avium* to TLR2. This interaction leads to bacteriostasis of *M. avium* in a MyD88-dependent way [10]. Even though the expression of TNF-α is also induced via TLR2-signalling, its role in growth restriction of *M. avium* is unclear [10]. IFN-γ is considered to be a key cytokine for killing of *M. avium* and its expression is promoted by IL-18 secreted by *M. avium*-infected human macrophages [11]. Phagocytosis of *M. avium* is supposed to be mediated via binding of the bacteria to a variety of receptors including complement receptors CR1, CR2, CR3, CR4, the mannosyl-fucosyl-receptor, the fibronectin receptor, the integrin receptor α(v)β_3_, and the transferrin receptor [12-15]. *M. avium* inhibits the acidification of the phagosome and the fusion of the phagosome with lysosomes [16,17].

Intracellular *M. avium* survives antibacterial activities such as nitric oxide and reactive oxygen species and the mechanisms leading to killing of *M. avium* are still unknown [18]. The cell wall structure is an important factor determining virulence of *M. avium*[19]. Thus, different colony morphotypes (smooth opaque, smooth transparent, rough) distinguishable on Congo Red plates display different degrees of virulence. Smooth transparent and rough colonies are considered to be more virulent than smooth opaque colonies [20,21]. The colony morphotype is associated with the glycopeptidolipid (GPL) composition [19]. By inducing the release of various pro-inflammatory cytokines such as IL-1, IL-6 or TNF-α, GPL modulate the immune response against mycobacteria [22].

Only relatively few virulence genes from MAH have been defined with respect to their role in infection. This is partly attributable to difficulties in generating MAH mutants. The major obstacle is the low transformation frequency if MAH is used as recipient. This also limits the efficiency of so far described random mutagenesis systems, such as the commercially available EZ-TN < KAN2 > Tnp Transposome from Epicentre. This Tn903-based system consists of a stable complex formed between the EZ::TN Transposase enzyme and the EZ::TN < KAN-2 > Transposon. It was used in MAA and MAH to analyse mechanisms of multidrug resistance and the role of GPL [23-25]. Another system for the generation of random mutants is based on transduction using temperature-sensitive phages containing a transposon with a selection marker [26,27]. In other mycobacterial species such as *M. tuberculosis* and *M. bovis* BCG linear recombination substrates have been applied to generate random as well as site-directed mutants [28-30]. This approach, however, so far has not been published for mutagenesis of MAH or MAA. With the present study we intended to explore the performance of illegitimate recombination of a linear recombination substrate for random mutagenesis of MAH.

## Methods

### Bacterial strains, amoeba, cell lines and growth conditions

Mycobacterial strains were grown in Middlebrook (MB) 7H9 broth (BD Biosciences, USA), supplemented with either 10% ADC (BD Biosciences) or 10% OADC (BD Biosciences) and 0.05% Tween 80 without shaking, and on MB 7H11 agar (BD Biosciences) at 37°C. *Escherichia coli* DH5α was used as host strain for plasmid pYUB854, a cosmid vector with a Hygromycin resistance (Hyg^r^) gene [31] and was cultured in/on Luria-Bertani broth and agar at 37°C. Antibiotics when required were added at the following concentrations: Kanamycin (50 μg ml^-1^) or Hygromycin (50 μg ml^-1^). For Congo Red plating agar media was supplemented with 100 μg ml^-1^ Congo Red. The *Acanthamoeba castellanii* strain 1BU group II [32] was cultivated in PYG medium (Proteose peptone-Yeast extract-Glucose [33]) at 28°C and passaged once per week. The human macrophage cell line THP-1 (DSMZ-No. ACC-16, DSMZ GmbH, Braunschweig, Germany) was maintained by passaging twice weekly in RPMI 1640 (GIBCO® Invitrogen, Darmstadt, Germany) supplemented with 10% foetal bovine serum (Bio Whittaker, Walkersville, MD, USA). Cells were cultured in BD Falcon^TM^ 75 cm^2^ trays (BD Biosciences) at 37°C and in 5% CO_2_. For human macrophages infection and washing, Iscove's Modified Dulbecco's Media (IMDM) (PAA laboratories, Austria) with 3% Human AB-serum (PAA laboratories) was used.

### Molecular biology techniques

All molecular biology techniques were carried out according to standard protocols [34] or according to the recommendations of the manufacturers of kits and enzymes. Primers were purchased from Metabion (Martinsried, Germany). Plasmid DNA was isolated with the QIAGEN Plasmid Mini Prep Kit (Qiagen, Hilden, Germany). Polymerase chain reaction (PCR) was performed with the DreamTaq Kit from Fermentas (St. Leon-Rot, Germany). Restriction enzymes were purchased from Fermentas. For elution of DNA fragments from agarose gels, the QIAquick Gel Extraction kit (Qiagen) was used. Ligation reactions were performed with the T4 DNA Ligase Kit from Fermentas. Genomic DNA from mycobacteria was isolated according to the protocol described in Sjöbring *et al.*[35]. Sequencing reactions were performed by using the Prism Big Dye FS Terminator Cycle Sequencing Ready Reaction Kit from PE Applied Biosystems (Darmstadt, Germany). Nucleotide sequence analysis was performed using the software packages MacVector™ 7.2.3 (Accelrys, Cambridge, UK) and Lasergene (DNASTAR, Inc., Madison, WI, USA).

For Southern blotting 2 μg of genomic DNA from *Mycobacterium* were digested with ApaI or SmaI, separated by electrophoresis in a 1% agarose gel and capillary transferred to positively charged nylon membranes (GE Healthcare, Buckinghamshire, UK) by following a standard protocol [34]. An 1818 bp region of the plasmid pYUB854 carrying the Hyg^r^ gene was amplified using the primer pair Hyg2K FW (5´-CAC CGT ACG TCT CGA GGA ATT CCT G-3´) and Hyg2K BW (5´-GCG TCG TGA AGA AGG TGT TGC TGA-3´) and the digoxigenin labeling Kit (Roche, Mannheim Germany). The labeled PCR-product was used as a probe and detection was carried out using anti-digoxigenin-AP conjugate and CDP-star (Roche) according to the manufacturers’ instructions.

Reverse PCR was applied to exactly locate the insertion sites of the Hyg^r^ gene in the mutants. 2 μg of DNA of each mutant was digested with the restriction enzyme ApaI or SmaI (which do not cut in the recombination substrate). The multiple sized DNA fragments were ethanol precipitated and then self-ligated by T4 DNA ligase enzyme, thus resulting in different sized circular DNA molecules. A PCR was then performed with primers [Hyg mut_1 (5´-AAC TGG CGC AGT TCC TCT G-3´) and Hyg mut_2 (5´-TCA GCA ACA CCT TCT TCA CGA-3´)] binding within the Hyg^r^ gene and oriented towards the unknown genomic MAH DNA located adjacent to the Hgy^r^ gene. Sequencing of the PCR products using the primers Hyg mut_1 and Hyg mut_2 followed by BLAST analysis of the sequences allowed the exact identification of the insertion sites of the recombination substrates.

For quantitative RT-PCR the mutants were grown in MB/ADC with 25 μg ml^-1^ of Hygromycin B to an OD_600_ of 2. The pellet of 10 ml of culture was resuspended in 4 ml of protoplasting buffer (15 mM of Tris–HCl pH 8, 0.45 M of Sucrose, 8 mM of EDTA) with 4 mg ml^-1^ Lysozyme. After incubation at 37°C for 45 minutes (min) the protoplasts were harvested by centrifugation and the pellets were resuspended in 1050 μl of the RLT buffer from the RNeasy Minikit (Qiagen) with 10.5 μl of ß-Mercaptoethanol. This suspension was transferred into tubes containing 25–50 mg of glass beads (0.5 mm, PeqLab, Erlangen, Germany) and shaken in the homogenizer Precellys 24 (PeqLab) for 45 sec at 6,500 g. The tubes were chilled on ice and centrifuged at 8,000 g for 5 min at 4°C. Then, 0.7 volume of absolute Ethanol was added to the supernatant and this solution was distributed onto two columns of the RNeasy Kit. The samples were further processed as described in the RNeasy manual. Residual DNA present in the RNA preparations was removed with the Kit Desoxyribonuclease I (DNaseI) RNase free from Fermentas. The M-MLV Reverse Transcriptase and Random primers from Promega (WI, USA) were used to transcribe cDNA from the RNA. The cDNA was then used to perform real time PCR with the Maxima^TM^ SYBR Green/Rox qPCR Master Mix 2x from Fermentas. Primers were (i) for gene MAV_1779: 5´-CTG CAG AAG AGC GTC TAC CC-3´ and 5´-CTC TGT TCG GAG GTC GTC AT-3´, (ii) for gene MAV_3129: 5´-GGT CAA GAC CAT CGA CGA CT-3´ and 5´-AGA TCA TGA ACG GCA CAA CA-3´, (iii) for gene MAV_4332: 5´-ATG GTC GAG CAG AGC ATC TGG-3´ and 5´-ATG GCG TCC ACG AAC CG-3´, (iv) for gene MAV_5105: 5´-GTT GTC GAG TTC ACC GGT CT-3´ and 5´-ATT CAC TCG GCG AAT ACC TG-3´ and (v) for 16S rRNA gene: 5´-GAG TGA GAA TGC AGG CAT GA-3´ and 5´-ACA CGG GTA CGG GAA TAT CA-3´. The ΔΔC_T_ method was used to calculate the relative expression of the gene of interest in the mutant in comparison to the mean of its expression in the other three mutants. Normalisation was obtained by measuring the expression of 16S rRNA gene as reference gene.

### Random mutagenesis by illegitimate recombination

1 μg of plasmid pYUB854 DNA was double digested with restriction enzymes StuI and SpeI Fast digest at 37°C for 30 min. The 2030 bp linear DNA fragment carrying the Hyg^r^ gene was gel-eluted after electrophoresis and 3–6 μg linear DNA fragment was transformed into *M. avium* strains by electroporation with the Biorad GenePulser apparatus applying 1000 Ω, 25 μF and 1.25 kV in 1 mm gap cuvettes. The preparation of electrocompetent cells and electroporation were performed using standard protocols [36]. Plasmid pMN437 was used as positive control for transformation [37]. Electroporated bacteria were incubated at 37°C for 24 hours (h) before plating on selective plates. Potential mutants were characterised by PCR amplifying a part of the Hyg^r^ gene [primers Hyg 2 K LC FW (5´-AGT TCC TCC GGA TCG GTG AA-3´) and Hyg 2 K LC BW (5´-AGG TCG TCC CGG AAC TGC TGC G-3´)], Southern blotting, reverse PCR (primers Hyg mut 1 and Hyg mut 2) and sequencing.

### Construction of a complemented derivative of mutant MAV_3128

Primers MAV3128_MV306_1 (5´-CGG TCT AGA CTA TGC CTA CCT GCT CTC-3´) and MAV3128_MV306_2 (5´-GCA GTT AAC CTA ATG CGG CTT GGC CAG-3´) were designed to amplify the gene MAV_3128 (3227 bp) plus 680 bp of upstream sequence of the wild type with *pfu* polymerase from Fermentas. The amplified product was cloned into the restriction sites XbaI and HpaI respectively of the integrative vector pMV306 [38]. The recombinant plasmid pFKaMAV3128 was transformed into *E. coli* DH5α by a method already described by Hanahan [39]. The plasmid pFKaMAV3128 was then introduced into competent cells of mutant MAV_3128 by electroporation. PCR analyses with the primer pair MAV3128_MV306_1 and 2 confirmed the presence of wild type gene in the mutant MAV_3128.

### Screening for virulence-mutants

#### Amoeba Plate Test (APT)

The APT was previously described [40]. In short, known concentrations of *Acanthamoeba castellanii* (1BU group II strain) diluted in PYG medium were spread on MB agar plates and these plates served as test plates. For control plates only PYG medium without amoeba was spread on MB agar plates. Plates were dried and incubated at room temperature. The next day series of tenfold dilution (1:10, 1:100, and 1:1000) in sterile water were prepared from cultures of the mutants and the *M. avium* 104 wild type (WT). 3 μl of undiluted culture and of each dilution were spotted onto the test and control agar plates. Plates were then incubated at 30°C for one week. Mutants showing reduced growth on test plates compared to the control plates were selected for further molecular characterisation.

#### Growth rate in broth cultures under pH stress

The growth rates of mutants and WT were compared in MB 7H9 broth with neutral pH (7) and under pH stress (pH 5). Cultures were inoculated to an initial OD_600_ of 0.02 to 0.03 and allowed to grow for two weeks. Three cultures per strain were inoculated. Growth of cultures was determined by measurement of OD_600_ of cultures and also by quantification of ATP with the luminescence-based Kit BacTiter-Glo^TM^ Microbial Cell Viability Assay (Promega). The luminescence was recorded as relative light units (RLU) with the microplate luminometer LB96V (EG & G Berthold). Mutants showing differences of growth pattern compared to the WT in both neutral medium and under pH stress conditions were considered for further molecular characterisation.

#### Congo Red plating

100 μl of 1:10^5^ and 1:10^6^ dilutions in sterile water of mutants, complemented strain and WT were spread in triplicate on MB agar plates supplemented with OADC and 100 μg ml^-1^ Congo Red. Plates were incubated for 2–3 weeks and observed for colony morphology. Mutants showing differences in colony morphology (white vs. red staining, transparent vs. opaque colonies, smooth vs. rough colonies) compared to the WT were considered for further molecular characterisation.

#### Induction of cytokine expression in THP-1 cells

Infection of the cell line THP-1 was performed in 24-well cell culture plates (TPP) with three to five wells per sample. A total of 200,000 cells per well of THP-1 were grown along with addition of phorbol-12-myristate-13-acetate (PMA, Sigma, Taufkirchen, Germany) (10 ng ml^-1^) and allowed to adhere to the surface of the plate well overnight at 37°C and in 5% CO_2_. Cells were then infected with mutants and WT at a multiplicity of infection (MOI) of 50 colony forming units (CFU). The supernatants were removed after 24 h and cytokines were quantified in appropriate dilutions of the supernatants by ELISA using the Human ELISA Ready to go Kits (Natutec, Frankfurt, Germany).

#### Intracellular survival in THP-1 cells

THP-1 cells were seeded, treated with PMA and infected as described above. The supernatants were removed after 4 h infection period and adherent cells were washed twice with RPMI 1640. The cells were then treated with 200 μg ml^-1^ of Amikacin (Sigma) for 2 h to kill the mycobacteria in the supernatant. After washing twice with PBS buffer (10 mM sodium phosphate, 126 mM sodium chloride, pH 7.2), 1 ml of medium supplemented with 5 μg ml^-1^ of Amikacin was added to each well. Samples for quantification of intracellular bacteria were taken at the end of the infection time after removal and killing of extracellular bacteria and then after 1, 2, and 4 days. For this, the cells were lysed in 1 ml of water at 37°C for 20 min and the mycobacterial DNA in the lysates was quantified by real-time PCR as described in Lewin *et al.*[41]. Additionally, 100 μl of 1:10^3^ dilution in sterile water of samples were plated in triplicate on agar plates supplemented with ADC for counting of CFU.

#### Intracellular survival in human monocytes

Human monocytes were isolated from buffy coats from healthy donors using Ficoll-Paque^TM^ Plus (GE Healthcare) and Percoll^TM^ (GE Healthcare) gradient centrifugation according to the manufacturer’s recommendations and as described in Sharbati *et al.*[42]. One million macrophages were seeded per well in 24-well cell culture plates, with three to five wells per sample per sampling point. Infection with mutants, complemented strain and WT, Amikacin treatment and sampling were done as described above for THP-1 cells infection, except that human monocytes were pre-activated with 100 U ml^-1^ of human IFN-γ (Invitrogen, Darmstadt, Germany) and 10 ng ml^-1^ of LPS (Sigma), IMDM was used for washing, the MOI for infection was 10 and the dilution of the samples for plating and counting of CFU was 1:500.

## Results and discussion

### Generation and genetic characterisation of *M. avium* mutants

Our aims were the establishment of a new method to mutagenise MAH and the identification of mutants potentially affected in virulence*.* The mutagenesis approach involved transformation of a recombination substrate by electroporation into MAH*,* and we therefore first identified clinical and environmental MAH strains applicable to electroporation. We considered a prior investigation of transformability to be necessary, because other authors had reported some clinical *M. avium* strains to be inaccessible to electroporation [43]. As proposed by Lee *et al.*[43], we chose a gfp-containing plasmid (pGFP: gfp cloned in vector pMV261 [38]) for transformation assays. We tested 14 clinical isolates and two soil isolates. Strain *M. avium* 104 was originally isolated from an HIV patient [44] and strains 2721/04, 10091/06, 10203/06, 4557/08, 4023/08, 3646/08, 3449/08, 3269/08, 2630/08, 2014/08, 772/08, 709/08, 528/08 were isolated from children with lymphadenitis. Strains 128 and 129 are soil isolates. Out of these 16 *M. avium* strains, five (104, 2721/04, 2014/08, 4023/08 and 528/08) could be transformed with pGFP. As the genome sequence from *M. avium* strain 104 is available in the genome data bases, simplifying a precise mutant description, we decided to concentrate on this strain for further analysis.

Our mutagenesis approach took advantage of the high rate of illegitimate recombination in slow growing mycobacteria [28,45] and their ability to take up linear DNA [29]. For selection purposes we chose the Hyg^r^ gene instead of also often used Kanamycin resistance gene (Km^r^), because the Hyg^r^ gene had been shown before to be superior to the Km^r^ gene especially for the transformation of other than laboratory strains [46]. The Hyg^r^ gene used for electroporation was flanked by plasmid DNA of 793 bp on one side and 238 bp on the other side. These flanking regions served as substrates for the illegitimate recombination. After electroporation of 3–6 μg of restriction fragment and selection on plates containing Hygromycin, about 1000 colonies could be obtained. Around ten percent of this number appeared on the negative control plates (no DNA added) and had to be considered spontaneously resistant. The positive controls (with 1–2 μg plasmid DNA) generated around 5–6 times more colonies than could be observed on the test plates. Transposon/transduction mutagenesis procedures have been reported to deliver around 1,000 to 3,500 mutants per mutagenesis procedure [19,23,24,27,47,48] which means that the efficiency or our method was below the efficiency of transposon/transduction systems. Taking into account the simple handling of our method we consider it nevertheless to be a good alternative to the currently applied methods for mutagenesis of MAH.

Fifty randomly chosen colonies from the sample plates were tested for insertion of the Hyg^r^ gene by performing a PCR using the primers Hyg 2 K LC FW and Hyg 2 K LC BW (data not shown). By this PCR 49 of the 50 colonies could be confirmed to carry an insertion of the Hyg^r^ gene in the genome. Additionally, Southern blots using a PCR fragment produced with primer pair Hyg2K FW and BW as probe were performed to verify if the insertions had occurred at different genome sites in different colonies (data not shown). Hybridising bands were obtained with the DNA from 20 colonies and confirmed independent insertion events.

**Figure 1:**
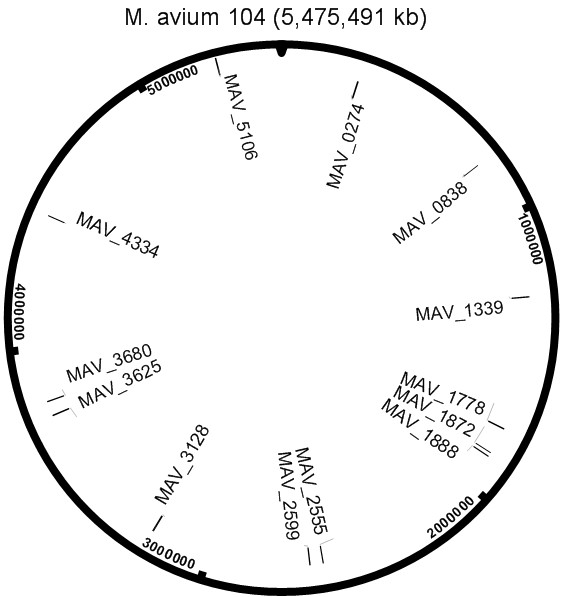
**Sketch showing randomly mutated genes distributed within the *M. avium *genome.** Genes location mapped on the genome after sequencing.

Inverse-PCR using the primers Hyg mut 1 and Hyg mut 2 followed by sequencing of the PCR products enabled us to identify the sites of insertion of the Hyg^r^ gene in 13 mutants. As shown in Figure 1, there were no hot spots for integration but the insertions were distributed within the whole *M. avium* genome.

**Figure 2:**
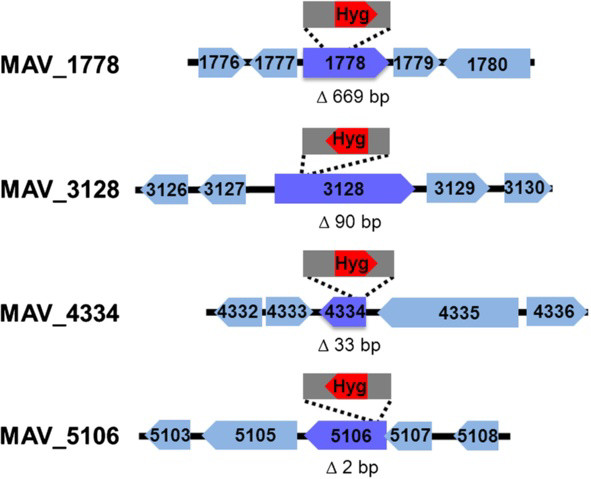
**Sketch illustrating the genetic characterisation of the mutants MAV_1778, MAV_3128, MAV_4334, and MAV_5106.** The sites of the insertion of the marker (Hyg^r^ gene) were identified by inverse PCR followed by sequencing of the eluted PCR products. The figure shows for four mutants the mutated gene (dark blue) with the site of insertion of the fragment (grey) carrying the Hyg^r^ gene (red) and the four genes located upstream and downstream of the mutated gene (light blue). Numbers in the arrows indicate the gene names. The direction of the arrows stands for gene direction. Gene sizes and distances between genes are approximations. Below the map of each mutant the size of the deletion generated as result of insertion of the marker is indicated.

The genetic characterisation of four virulence-associated mutants is shown in Figure 2. The integration events were accompanied by deletions in all 13 mutants. The smallest deletion had a size of 2 bp, the largest one of 669 bp. All insertions were located within coding regions. Only in one mutant more than one gene was affected by the insertion. In 12 of the 13 mutants the linear recombination substrate had been completely inserted and in one mutant the inserted fragment had been shortened at both ends. The sequences next to the inserted fragment showed no special structure or nucleotide sequences.

Both, the random distribution of insertion sites and the low rate of large deletions affecting more than one gene are benefits of our method. Contrary to our experience with MAH, Collins and colleagues [49] observed more clustered insertions and deletions of up to 12 genes by mutagenising *M. bovis* with a DNA fragment carrying a Kanamycin resistance gene by illegitimate recombination. It would be interesting to find out the reasons for these differing outcomes. Are the specific parameters of the illegitimate recombination events species-specific or does the composition of the recombination substrate play a more important role?

In favor of a straight forward procedure, we concentrated our further efforts on those mutants, which fulfilled the following requirements: - an insertion in the middle of the coding region of a gene, – mutation of only one gene and - mutation of a single copy gene. After applying these criteria, eight mutants (see Table 1 for mutated genes and their functions) were selected for phenotypic analysis.

**Table 1:** **Mutated *M. avium *genes and their functions**

**Mutated Gene**	**Function of the gene**
MAV_2555	Short-chain dehydrogenase/reductase SDR
MAV_1888	Hypothetical protein
MAV_4334	Nitroreductase family protein
MAV_5106	Phosphoenolpyruvate carboxykinase
MAV_1778	GTP-Binding protein LepA
MAV_3128	Lysl-tRNA synthetase (LysS)
MAV_3625	Hypothetical protein
MAV_2599	Hypothetical protein

### Phenotypic characterisation of MAH mutants

Since virulence is regulated on many different levels we applied more than one screening test (as for example intracellular multiplication) to identify a greater spectrum of relevant virulence-associated genes. We searched for phenotypic assays allowing a fast screening of our mutants and not requiring special and expensive equipment. The selected tests should monitor changes in (i) cell wall composition (plating on Congo Red Agar), (ii) resistance towards low pH, (iii) amoeba resistance, (iv) induction of cytokine secretion by infected macrophages and (v) intracellular survival and growth in human macrophages.

#### Colony morphology and Congo Red staining characteristics

The occurrence of different colony morphotypes is an eye-catching feature of *M. avium* including MAH and has attracted attention also because it is associated to virulence [19,24,50,51]. The colony morphology is influenced by the composition of the cell wall, which is a major determinant of mycobacterial virulence [52-54]. Congo Red, a planar hydrophobic molecule can bind to diverse lipids and lipoproteins and is thus applicable for the detection of changes in cell wall composition [54-56]. Upon plating of MAH on Congo Red agar plates, smooth transparent, smooth opaque and rough colonies as well as red and white colonies can be distinguished. Appearance of the three morphotypes smooth opaque, smooth transparent and rough was also confirmed for strain 104 [21]. While the opaque-transparent switch is reversible, the rough phenotype results from irreversible deletion of cell envelope glycopeptidolipid genes and is irreversible [24,51].

TLC (Thin Layer Chromatography) analysis of the different morphotypes from strain 104 has been performed by Torelles [21]. They also analysed the sugar composition of the glycopeptidolipids (GPL) by gas chromatography–mass spectrometry (GC–MS) analysis. They found that the smooth opaque and smooth transparent colonies formed similar GPL and both expressed besides the nsGPL (ns: non-specific) the ssGPL (ss:serovar specific) of serovar 1. However, the ssGPL was absent in the rough morphotype, which had a strong band of the nsGPL. A band in the lipopeptid region devoid of sugars was present in the smooth transparent morphotype and the rough morphotype but lacking in the smooth opaque morphotype. The sugar composition of all morphotypes showed the typical profiles related to ns and ssGPL of serovar 1, only in the rough morphotype 6-deoxytalose and 3-*O*-methyl-6-deoxytalose were missing.

The transparent colony variant grows better in macrophages and animals compared to the opaque variant. Moreover, white transparent colonies survived better in macrophages than red transparent colonies [19,24,50,51,56]. These differences in intracellular survival may be caused by variations in the cytokine response towards infection by different morphotypes. The smooth opaque morphotype has been shown to induce higher levels of secretion of IL-1α, IL-1β and TNF-α by human blood-derived monocytes compared to the smooth-transparent morphotype [57]. Variation in cytokine response upon infection with either smooth-opaque or smooth-transparent *M. avium* was also reported upon infection of human microglia cultures [58].

**Figure 3:**
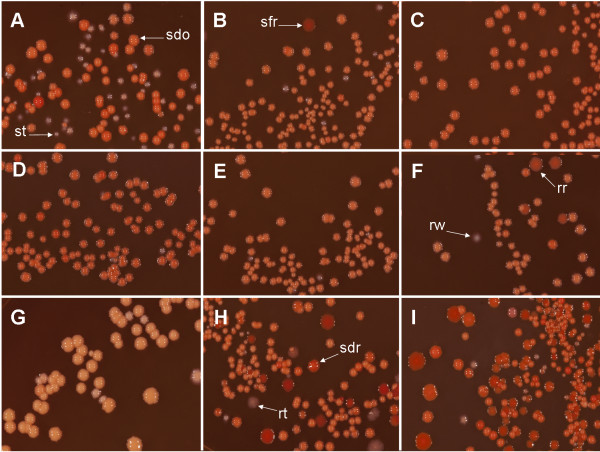
**Colony morphology upon plating on Congo Red agar plates.** Well-grown broth cultures of all strains were diluted 1:10^6^ and 100 μl plated in triplicate onto Middlebrook agar with OADC containing 100 μg ml^-1^ Congo Red. Plates were incubated on average for three weeks. The arrows point to smooth-domed-opaque (sdo), smooth-flat-red (sfr), smooth transparent (st), rough red (rr) and rough transparent (rt) colonies. **A**: WT; **B**: mutant MAV_2555; **C**: mutant MAV_1888; **D**: mutant MAV_4334; **E**: mutant MAV_5106; **F**: mutant MAV_1778; **G**: mutant MAV_3128; **H**: mutant MAV_3625; **I**: mutant MAV_2599.

The colony morphology of the WT and the mutants upon plating on Congo Red Agar is shown in Figure 3. The WT (Figure 3 A) mainly formed smooth-domed-opaque (sdo) colonies along with smooth-transparent (st) colonies. Mutant MAV_2555 showed the same morphologies, but additionally smooth-flat-red (sfr) colonies were visible (Figure 3 B). Relatively few smooth-transparent and rough colonies occurred in mutant MAV_1888 (Figure 3 C), MAV_4334 (Figure 3 D) and MAV_5106 (Figure 3 E). Mutant MAV_4334 (Figure 3 D) showed a higher variation with respect to the intensity of red color of smooth-domed-opaque colonies. Mutant MAV_1778 showed a very high degree of variability displaying red-rough (rr) and smooth-flat-red colonies additionally to the smooth-domed-opaque, smooth-transparent and rough-white (rw) colonies (Figure 3 F). The colonies generated by mutant MAV_3128 (Figure 3 G) were in average larger in size and the smooth-opaque colonies appeared paler than in the WT. Also, the edges of these colonies were more irregular. Some red-rough colonies were also visible. The most multifaceted image was displayed by mutant MAV_3625. This strain generated smooth-domed-opaque, smooth-domed-red (sdr), smooth-flat-red, smooth-transparent and rough-transparent (rt) and red-rough colonies (Figure 3 H). A high proportion of red colonies (smooth-domed-red, smooth-flat-red, red-rough) was generated by mutant MAV_2599 (Figure 3 I) additionally to smooth-opaque and smooth-transparent colonies. This mutant produced only few rough (rough-transparent, rough-red) colonies. Altogether, we observed a high frequency and intensity of morphological changes in the mutants pointing to involvement of the mutated genes in the composition of cell wall structure. Since studies by different authors have related colony morphotype to virulence it would be of interest to investigate in further experiments if and to which degree the different colony types are stable and differ in their virulence.

#### pH-resistance

**Figure 4:**
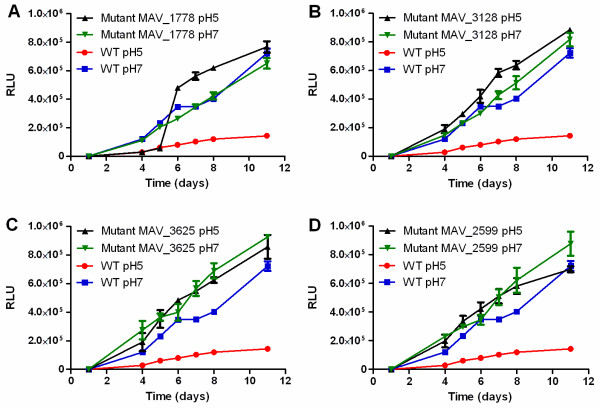
**Resistance towards pH stress.** The bacteria were grown in Middlebrook 7H9 broth with OADC at pH 7 and pH 5 during 11 days; the ATP content was recorded by quantification of the amount of ATP in the cultures. The amount of ATP is represented as RLU (relative light units). **A**: WT and mutant MAV_1778; **B**: WT and mutant MAV_3128; **C**: WT and mutant MAV_3625; **D**: WT and mutant MAV_2599.

The intraphagosomal pH of *M. avium*-containing phagosomes decreases to pH 5.2 in activated macrophages [59]. We therefore investigated the pH-resistance of the mutants compared to the WT by inoculating them into MB broth at pH 5 and pH 7 and measuring the growth during 11 days at 37°C by means of OD measurement and ATP quantification. ATP measurement represents a much more sensitive method than the OD measurement. Additionally, the OD of a culture not only depends on cell number but also on the size of the cells, their morphology and the degree of clumping of the cells. For these reasons, ATP measurement was reported to be a more reliable method for quantification of mycobacteria in broth culture [41]. As shown in Figure 4, the WT grew better at neutral pH than at low pH. After 11 days of growth in neutral medium, it generated 722,491 RLU (relative light units), while in medium with acidic pH only 143,082 RLU were achieved. The mutants MAV_2555, MAV_1888, MAV_4334 and MAV_5106 showed a similar growth pattern as the WT, both in neutral and acidic pH (data not shown). The mutants MAV_1778 and MAV_3128 grew similar as the WT at neutral pH; however, at low pH these strains enhanced their growth rate even above the level reached at neutral pH (Figure 4 A and B). While the mutant MAV_3128 showed enhanced growth in comparison to the WT at low pH already at day 1, the mutant MAV_1778 showed an identical growth rate as the WT at low pH until day 5 and then showed strongly enhanced growth. The mutants MAV_3625 and MAV2599 grew better than the WT at pH 7 and were able to maintain this growth rate at pH 5 (Figure 4 C and D). In summary, the mutations either had no influence on the survival under pH stress conditions or improved resistance towards pH stress.

#### Amoeba plating test

Free-living amoebae are known to host environmental mycobacteria including *M. avium*, which are able to survive in *Acanthamoeba* trophozoites as well as in the exocysts [4,60,61]. Growth in *Acanthamoeba* was associated with subsequently enhanced virulence in infection experiments with mice [62]. Since some virulence mechanisms are employed by amoeba-resistant bacteria to survive in amoebae as well as in macrophages [4,63-65], amoebae have been used as test systems for determination of bacterial virulence factors [40,63,66]. An *Acanthamoeba castellanii* agar plate assay was developed and successfully employed for screening of mutants of *Legionella pneumophila*[40]. We adapted this APT to fit the growth conditions (medium, temperature, duration) of *M. avium* and tested the eight mutants in comparison to the WT. After incubation for five to seven days at 28°C, the WT formed colonies even if the cultures were diluted 1:10^3^ before being dropped on the lawn of amoebae. The growth of some mutants was more strongly affected by the amoebae but a differentiated evaluation of the impact of the various mutations on survival in the amoebae was not possible (data not shown). The APT thus was not sensitive enough to reveal differences in the capacity of the mutants to survive within the amoebae. This was surprising, because the APT has proven to be an efficient tool for the identification of virulence genes in *L. pneumophilae*[40]. There are several possible explanations for this discrepancy. Amoebae are the most important habitat of *Legionella*, while *M. avium* is not dependent on the presence of amoebae for survival and distribution. As a consequence, *Legionella* might have evolved more important virulence factors interacting with amoebae. Another possible explanation may result from the differences in the generation times of *L. pneumophilae* and *M. avium*. *L. pneumophilae* is a fast-growing bacterium forming clearly visible colonies few days after plating, while the slow-growing *M. avium* 104 requires two weeks to generate colonies of comparable size. This time span may be too long to maintain the amoebae as trophozoites actively interacting with the mycobacteria. In conclusion, we estimate the APT to be of only little value for the detection of virulence genes of slow-growing mycobacteria.

#### Induction of cytokine secretion

**Figure 5:**
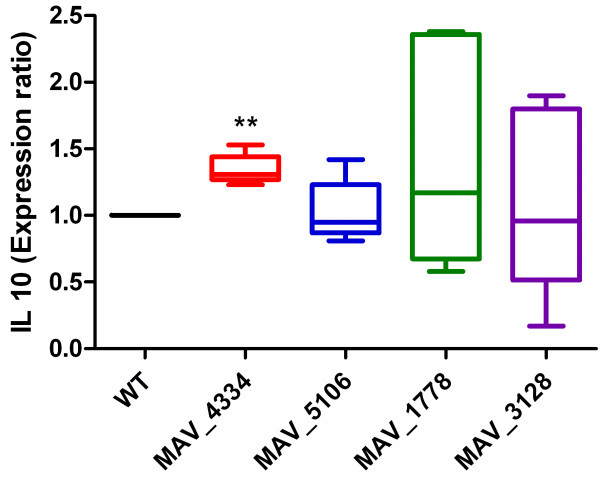
**Induction of IL-10 cytokine secretion by infected macrophages.** THP-1 cells (2.0x10^5^) were infected (MOI 50) with mutants and WT. After 24 hours cytokines from supernatants were measured by ELISA. When compared to WT a P value <0.01 (two-tailed, unpaired Mann–Whitney test) was considered very significant (**).

The innate immune recognition by phagocytic cells mediates cellular activation enabling killing of the bacteria and the production of pro- and anti-inflammatory cytokines. The signaling cascade is mainly initiated by binding of *M. avium* components to TLR2 followed by recruitment of the MyD88 adaptor molecule and the activation of NFκB and MAP kinases. This chain of events ends with the induction of inflammatory cytokines [10] controlling macrophage activation and granuloma formation. We monitored the induction of cytokine expression of THP-1 macrophages by the WT and the mutants in order to evaluate their ability to stimulate the immune signaling. To this aim we quantified the secretion of selected cytokines: the pro-inflammatory cytokines TNF-α, IL-1β and the anti-inflammatory cytokine IL-10. Five independent experiments were normalised for WT (expression ratio 1) to determine the expression ratio for the mutants in comparison to WT. While results for TNF-α and IL-1β were not significantly different as compared to WT, IL-10 was significantly (P <0.007) up-regulated for mutant MAV_4334 (Figure 5). IL-10 can inhibit the production of inflammatory cytokines such as TNF-α in monocytes pre-activated by IFN-γ and LPS [67,68] and therefore plays an important role in the immune response.

#### Intracellular survival

**Figure 6:**
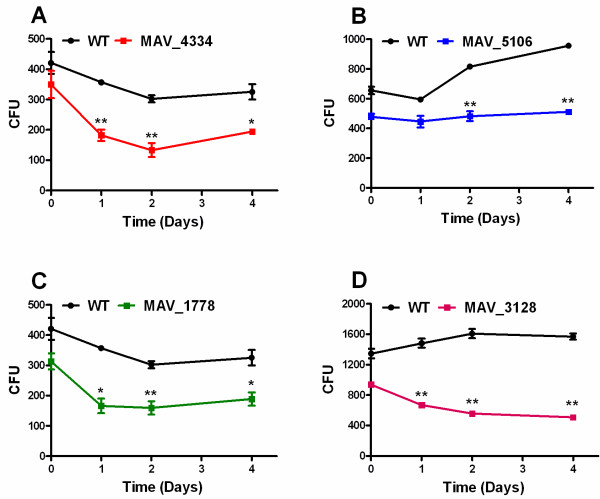
**Intracellular survival of mutants compared to WT in human monocytes.** Human blood monocytes (1.0x10^6^) from healthy volunteers were infected (MOI 10) with mutants and WT. Intracellular bacteria were quantified after 4 hour of infection, and after 1, 2, & 4 days. The monocytes were lysed in 1 ml of sterile water and 100 μl of 1:500 dilution in sterile water of sample were plated on Middlebrook agar plates supplemented with ADC for CFU counting. **A**: WT and mutant MAV_4334; **B**: WT and mutant MAV_5106; **C**: WT and mutant MAV_1778; **D**: WT and mutant MAV_3128. Statistical analysis was done using a two tailed, paired Student’s t test. When compared to wild-type a P < 0.05 was considered significant (*) and a P < 0.01 very significant (**).

The ability to survive and even replicate inside the phagosomes of macrophages is an important virulence factor of mycobacteria and was therefore included in our screening options. Infection experiments with macrophages give information on the early host response to mycobacterial infections [69]. Different types of macrophages or monocytic cells have been employed to assess mycobacterial virulence and among these the human macrophage-like cell line THP-1 has proven a suitable system for virulence testing [69,70]. It was shown that THP-1 cells are similar to primary human monocyte-derived macrophages with respect to their ability to take up mycobacteria and limit their growth [71]. We infected THP-1 cells that had been differentiated by PMA with the WT and the mutants. Intracellular mycobacteria were measured by quantitative real-time PCR and CFU by plating. Survival of mutants in THP-1 cells was not consistently different if compared to the WT (data not shown). More significant differences were obtained when using human blood monocytes for the infection experiments. The growth of mutant MAV_4334, MAV_1778 and MAV_3128 was affected the most in human monocytes (Figure 6). They were reduced significantly for the first two days (P < 0.05 to P < 0.01). Mutant MAV_4334 and MAV_1778 (Figure 6 A and C) were almost reduced to half during the first two days. As shown in Figure 6 D, mutant MAV_3128 had the highest significant (P < 0.001) difference in growth as compared to WT, which had survived better during this time period. The mutant MAV_5106 largely differed from other mutants and during four days of infection had shown constant survival (Figure 6 B). The capacity of mutant MAV_5106 to survive better in macrophages suggests that it may be characterised by a higher virulence as compared to the other mutants. Tateish *et al.*[70] compared the virulence of different *M. avium* isolates in humans, immuno-competent mice and THP-1 cells. They found that the strain causing the most serious disease in humans and the highest bacterial load in mouse lungs also grew better in THP-1 cells than the other strains tested. According to this, the mutants MAV_4334, MAV_1778 and MAV_3128 may display reduced virulence and the corresponding genes may represent virulence-associated genes.

### Evaluation of the screening procedure

We have employed five screening methods (colony morphology, pH stress resistance, amoeba resistance, cytokine induction, intracellular survival) to select mutants affected in virulence-related traits. Two mutants (MAV_4334 and MAV_3128) responded differently from the WT in four of these five screening tests and two mutants (MAV_5106 and MAV_1778) reacted differently in three screening tests. The most prominent differences were exhibited by mutant MAV_3128. The other mutants either did not show any differences compared to the WT or reacted differently in only one or two tests.

The insertions in mutants MAV_4334, MAV_5106, MAV_1778 and MAV_3128 have been mapped and the structure of the mutated regions has been analyzed on nucleotide level. In all cases only one gene has been mutagenised. The insertions are located in the genes MAV_4334 (nitrogenase reductase family), MAV_5106 (phosphoenolpyruvate carboxykinase), MAV_1778 (GTP-binding protein LepA) and MAV_3128 (lysyl-tRNA synthestase LysS).

Phosphoenolpyruvate carboxykinases (PEPCK) catalyse the reversible decarboxylation and phosphorylation of oxaloacetate to form phosphoenolpyruvate. Mutations of the PEPCK gene from *M. bovis* BCG are characterised by attenuated virulence and reduced survival in macrophages [72]. The PEPCK gene from *M. tuberculosis* was shown to be required for replication in murine bone marrow macrophages and mice [73].

The LepA protein from *M. tuberculosis* possess GTPase activity. Bacterial GTP-binding proteins play a role in regulation of ribosomal function and cell cycle, modulation of DNA partitioning and DNA segregation [74]. In *Helicobacter pylori* LepA is important for growth at low pH and may play a role in infection [75].

The *lysS* gene from *M. avium* is 81% homologous to the *lysX* gene from *M. tuberculosis*. LysX from *M. tuberculosis* is required for synthesis of lysinylated phosphatidylglycerol. A LysX mutant was shown to be sensitive to cationic antibiotics and peptides, to be more lysosome-associated and to display defective growth in mouse and guinea pig lungs [76].

So far, nothing is known about the role of the nitrogenase reductase family protein for growth and pathogenicity of mycobacteria and answering this question will be one of our future aims.

In summary, by analysing 50 random mutants, we uncovered four genes from MAH to play a role in the interaction with host cells and thus in virulence. The homologues of three of the four genes were shown to contribute to virulence in other bacterial species, which supports the significance of our screening procedure.

### Mutant complementation and evaluation of polar down-stream effects

To prove that the phenotypes of the mutants were indeed a cause of the inactivation of the mutated genes, we aimed at complementing the mutants by introducing the intact genes by electroporation. Only the transfer of gene MAV_3128 into the respective mutant was successful. Mutant MAV_3128 had shown the strongest and most different phenotypic changes in comparison to wild-type among the eight tested mutants in almost all the phenotypic tests. A complementation is best performed if the copy number of gene transcripts generated by the complementing gene narrows the copy number in the wild-type. We therefore used a plasmid for cloning (pMV306) that integrates once in the genome of the mutant and included the upstream region of *MAV_3128* to most likely cover the promoter of the gene. This upstream region had a size of about 680 bp and the gene *MAV_3127*, which is located upstream of *MAV_3128*, has an orientation in opposite direction of *MAV_3128* (see Figure 2). Therefore it was expected that the upstream region will contain the promoter sequence of the *MAV_3128* gene. Thus a 3907 bp DNA fragment was cloned into the integrative vector pMV306. The resulting recombinant plasmid pFKaMAV3128 was successfully transformed into the mutant MAV_3128 to generate the complemented strain *MAV3128Comp*.

**Figure 7:**
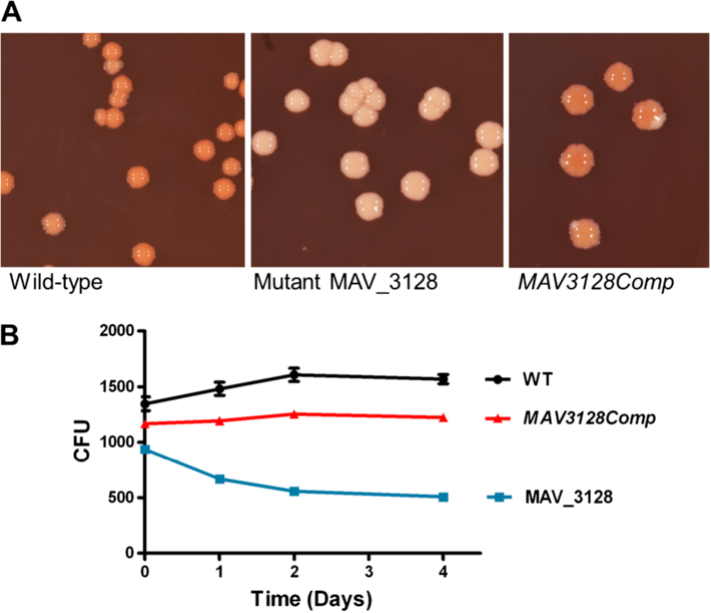
**Phenotype of the complemented strain *MAV3128Comp *compared to mutant MAV_3128 and WT. A**: Colony morphology on Congo Red plates. **B**: Intracellular survival in human blood monocytes.

Selected phenotypic tests (plating on Congo Red Agar and intracellular survival) were repeated with the complemented strain. Upon plating on Congo Red agar (Figure 7 A), the pale colour of mutant MAV_3128 could no longer be seen in *MAV3128Comp*, except some pale corners in colonies. This may indicate the loss of the plasmid in absence of selection pressure. The intracellular survival experiment has also conclusively indicated a reversal of the mutation. The complemented strain showed more similar growth tendency towards wild-type strain than towards the mutant (Figure 7 B). In conclusion we successfully complemented the mutant MAV_3128 by introducing the intact gene proving that the phenotype of mutant MAV_3128 was indeed caused by the inactivation of gene *MAV_3128* and not by a second line mutation.

Since introduction of the intact genes into the other three mutants failed we additionally investigated the occurrence of polar effects in the four mutants by quantitative RT-PCR. As polar effects most probably will have an impact on genes which are located downstream of the mutated gene and exhibit the same orientation, we quantified expression of genes *MAV_1779* (in mutant MAV_1778), *MAV_3129* (in mutant MAV_3128), *MAV_4332* (in mutant MAV_4334) and *MAV_5105* (in mutant MAV_5106) by qRT-PCR. The 16S rRNA gene was used as reference gene. The ΔΔC_T_ method was used to calculate expression of the gene in the corresponding mutant compared to the mean expression in the other three mutants. The expression levels measured were: *MAV_1779* (in mutant MAV_1778): 2.1 fold, *MAV_3129* (in mutant MAV_3128): 1.1 fold, *MAV_4332* (in mutant MAV_4334): 1.0 fold and *MAV_5105* (in mutant MAV_5106): 1.4 fold. In three of the four mutants, the expression of the down-stream genes transcribed in the same direction was not or only slightly changed. Only in mutant MAV_1778 a two-fold expression of gene *MAV_1779* was observed. We conclude that with one exception no relevant polar effects could be observed.

## Conclusions

Our study proposes a well-functioning method to randomly mutagenise MAH, by illegitimate recombination, genetically characterise the mutations to the nucleotide level and screen the mutants with simple phenotypic tests providing information about virulence-associated features.

## Competing interests

The authors declare that they have no competing interests.

## Authors’ contributions

Conceived and designed the study: FAK and AL. Carried out the Laboratory work: FAK, AK, EK and RK. Manuscript drafted: FAK and AL. All authors read and approved the final manuscript.
